# Evaluation on Genotoxicity and Teratogenicity of Aqueous Extract from *Cyclocarya paliurus* Leaves

**DOI:** 10.1155/2014/498134

**Published:** 2014-03-18

**Authors:** Lihong Deng, Jiandu Lei, Jing He, Jing Liu, Luying Wang, Rui Zhang, Xinhua Liu, Yun Liu

**Affiliations:** ^1^Beijing Key Laboratory of Lignocellulosic Chemistry, Beijing Forest University, Beijing 100083, China; ^2^Beijing Key Laboratory of Bioprocess, College of Life Science and Technology, Beijing University of Chemical Technology, Beijing 100029, China; ^3^Hunan Cyclocarya paliurus Technology Development Co. Ltd, Shuiling, Hunan 411100, China

## Abstract

Tremendous attentions have been attracted to the foods labeled with natural, green, organic, and nuisanceless conception of healthy diet. Therefore, it is of great significance to establish relative defining guidance for safe assessment of botanicals. *Cyclocarya paliurus* (Batal.) Iljinsk (family Cyclocaryaceae), called sweet tea tree, is a well-known edible and medicinal plant, which has been widely used in China as drug formulation for the treatment of hypertension and diabetes. Despite its benefits, no reports have been described on the safe assessment of *C. paliurus* leaves aqueous extract. In this study, we have conducted the genotoxicity assay (including Ames test, bone marrow polychromatic erythrocyte micronucleus test, and sperm abnormality test in mice) and traditional teratogenicity assay in rats (maternal toxicity, embryo toxicity, and teratogenicity test) to assess the genetic and teratogenic safety of aqueous extracts from *C. paliurus* leaves. Results of each assay show that the highest dose of *C. paliurus* leaves aqueous extract is considered relatively nonmutagenic and nonteratogenic, revealing that *C. paliurus* leaves possess safety and quality as a functional additional ingredient in food.

## 1. Introduction

*Cyclocarya paliurus*, an endemic plant in China and the sole species in the genus* Cyclocarya* Iljinskaja (Juglandaceae), is mainly found in the cloudy and foggy high-mountainous regions in southern China [1, 2], and possesses activities of anti-microorganisms, enhancement of mental efficiency and immunomodulation [3–9]. It has also been revealed that the chemical constituents of this plant contain protein, polysaccharides [3, 5, 10–12], triterpenoids, flavonoids, steroids, saponins, and phenolic compounds [13–16].

The leaves of* C. paliurus* have been used for drug formulations in traditional Chinese medicine, as well as an ingredient in functional foods in China [5] or dietary supplements [3]; they have even been a food resource for maritime people for a long time [13], which are processed as tea products, the first FDA-approved health tea in China in 1999 [17], and consumed as a beverage in Chinese daily life. The recommended human dose of this plant's leaves is 0.133 g/kg·bw. With the developing concerns about healthy diet, the natural green plant and its derived products have turned to be new favorite products in food industry [18]. However, in modern society, because of the undesirable adverse health effects appearing due to the use of plant-based outcomes, it has been clearly realized that “natural” is not equal to “safe” [19]. Recently, there have been reports that some traditional medical plants cause genotoxicity or cancer. The botanicals and herbal food supplements, which are generally recognized as safe, need to be set up legislative frameworks and guidance for risk assessment forwardly [20]. Delightedly, several organizations have undertaken the work and achieved results [21, 22]. However, to our knowledge, little information has been critically given on the issue of safety, such as the toxicity of* C*.* paliurus*.

The acute and subacute toxicity test of aqueous extracts from* C. paliurus* leaves in mice and rats had been conducted in our group. The result shows that the highest tested dosage of 40.0 g/kg·bw, equivalent to 300-fold of human daily intake (EDI), was not associated with any toxicity concern. Therefore, the aim of this study was focused on examining whether the aqueous extracts of* C*.* paliurus* leaves cause genotoxicity and teratogenic toxicity or not. Ames test, mouse bone marrow polychromatic erythrocyte micronucleus test, and mouse sperm abnormality test were conducted to evaluate the genetic safety of aqueous extracts of* C*.* paliurus* leaves. This study may give further information about the edible security of* C*.* paliurus* leaves as a potential source for functional food ingredient with multibioactivities.

## 2. Materials and Methods

### 2.1. Materials and Tested Animals

Leaves of* C. paliurus* were provided by Hunan* Cyclocarya paliurus* Technology Development Co. Ltd, Suining county, Hunan province, China, with recommended human dose of 0.133 g/kg·bw. The air-dried leaves are light brown and were milled into powder using 0.2 mm sieve. The milled leaves were extracted with distilled water for 30 min at 85°C at a ratio of water to leaves of 10 : 1 (mL : g). The extraction was performed duplicate and all extracts were combined and concentrated to a concentration equivalent to 2 g leaves per millimeter liquid.

The SPF grade Kunming mice, SD rats, and the fodder were provided by Dongchuang Animals Experiment Technology Services, Kaifu district, Changsha (experimental animal production license number: SCXK (Xiang) 2009-0012, experimental animal permission license number: SYXK (Xiang) 2010-0010). The test animals were developed in the shielding environment of temperature of 22~24°C and humidity of 52~56%.

### 2.2. Bacterial Reverse Mutation Test (Ames Test)

Ames tests were carried out in histidine auxotrophic* Salmonella typhimurium *strains TA97, TA98, TA100, and TA102. Test groups received five doses of the aqueous extract of* C. paliurus *leaves, equivalent to the doses of 5000 *μ*g/plate, 1000 *μ*g/plate, 200 *μ*g/plate, 40 *μ*g/plate, and 8 *μ*g/plate, in the presence or absence of rat liver microsomal enzyme (S-9) as in vitro metabolic activation system. The following controls were conducted simultaneously: spontaneous reverse mutation test for all strains, solvent for all strains, positive for TA97+S9, TA98+S9, and TA100+S9 of 10 *μ*g/plate 2-AF, positive for TA97-S9 and TA98-S9 of 0.2 *μ*g/plate 9-fluorenone, positive for TA100-S9 of 1.5 *μ*g/plate NaN_3_, positive for TA102+S9 of 50.0 *μ*g/plate 1,8-mitoxanone, and positive for TA102-S9 of 0.3 *μ*g/plate MMC, respectively. It was defined as positive when the number of reverse mutant colonies of the tested substance exceeded over twice as much as that of the spontaneous reverse mutation test or reflected a dose-response relationship. The whole test was repeatedly carried out twice under the same conditions.

### 2.3. Bone Marrow Micronucleus Test in Mice

The test was conducted by method of oral gavage twice interval of 24 h. 50 Kunming mice with weight of 25~30 g were divided randomly into 5 groups of 10 mice each, 5 males and 5 females. The test groups were administered 0.2 mL differently diluted aqueous extracts liquid of per 10 g body of* Cyclocarya paliurus* leaves, equivalent to the dose of 10.0 g/kg·bw (high dose level), 5.0 g/kg·bw (middle dose level), and 2.5 g/kg·bw (low dose level), respectively. 40 mg/kg·bw of cyclophosphamide was used as positive controls and distilled water as negative controls. Six hours after last oral gavage, the mice were sacrificed by cervical dislocation. The sternum marrow was taken to dilute with calf serum and smear. The slides were fixed with methanol, washed with distilled water, and stained with Giemsa. A total of 5000 polychromatic erythrocytes (PCE) calculated from 1000 PCEs per animal were analyzed to count the total number of micronucleated polychromatic erythrocyte (MNPCE) under microscope. The micronucleus incidence was counted by MNPCEs/PCEs (‰). A total of 1000 polychromatic erythrocytes (PCE) calculated from 200 PCEs per animal were used to determine the index of PCEs/NCEs (norm chromatic erythrocytes).

### 2.4. Sperm Deformity Test in Mice

25 male Kunming mice with weight of 25 g ~30 g were divided randomly into 5 groups, including three dosages test groups, one negative control group and one positive group, 5 mice each. At intervals of 24 h, the test groups received oral gavages of aqueous extracts of* Cyclocarya paliurus* leaves at three doses, 10.0 g/kg*·*bw (high dose level), 5.0 g/kg·bw (middle dose level), and 2.5 g/kg*·*bw (low dose level), respectively. 40 mg/kg*·*bw of Cytoxan was used as positive controls and distilled water as negative controls. The oral gavage was conducted continuously for 5 days. On the thirtieth day after the last gavage, the mice were sacrificed by cervical dislocation to get the bilateral epididymis. The sperms were spread on a slide glass and stained with eosin. A total of 1000 structurally completed sperms were scored for each animal under light microscope to evaluate the incidence of sperm deformity. A positive result for the sperm deformity in each test group was determined by a dose-dependent increase in the number of abnormal sperms or a reproducible positive reaction and statistical significance at one or more dosages.

### 2.5. *Cyclocarya paliurus* Teratogenicity Test

120 sexual maturity female SD rats and 60 male SD rats were employed to receive the test. One female and one male were kept in the same cage. Every morning the finding of vaginal plug or sperm in the female vagina identified the copulation of the rats and the day was regarded as the zero-day of fertilization. If the copulation did not occur within 5 days, the male rat would be replaced. The 60 detected pregnant rats were weighed, numbered, and divided randomly into 5 groups, including three dosages test groups, one negative control group and one positive group, 12 rats each group. In the gestation of 7 to 16 days, the three test groups received oral gavage of 1.0 mL differently diluted aqueous extracts of* Cyclocarya paliurus* leaves per 100 g body weight at interval of 24 h, equivalent to the dose of 20.0 g/kg·bw (high dose level), 10.0 g/kg·bw (middle dose level), and 5.0 g/kg·bw (low dose level), respectively. The oral gavage of 1.0 mL aspirin/100 g·bw was used as positive controls, equivalent to the dose of 0.27 g/kg·bw, and distilled water as negative controls. Each rat was provided enough diet. The oral gavage amount was adjusted based on the body weighing on the 0, 7th, 12th, 16th, and 20th day of gestation. On the 20th day, the pregnant rats were sacrificed and punched to obtain the ovaries and matrixes. The corpora lutea, implantation, absorbed fetus, dead fetus, and live fetus were counted. Ovaria, wombs linking to placenta, and placentas were weighed, respectively. The live fetus sexuality was identified one by one and the body weight, body length, and tail length were determined and checked whether appearance dysmorphia was occurred or not. After that, half of live fetuses (odd or even) of each pregnant rat were fixed in 95% (v/v) ethanol for 3 weeks, washed with running water, and immersed in the 2 g/100 mL potassium hydroxide for 72 hours. When the fetuses became hyaline, they were stained with alizarin red for 48 hours with shaking twice a day till the skulls became red. Then the fetuses were soaked in transparent liquid A for 2 days and transparent liquid B for 3 days in succession till the bones were incarnadined while the soft tissue faded. The overall examination and skeletal survey were conducted by the stereo-microscope. The other half live fetus was fixed by immersing in Bouin's for 2 weeks for viscous examination.

### 2.6. Data Statistical Analysis

The data were analyzed by Spss11.0. The homogeneity of variance of the data was tested firstly. If it was homogeneous variance, single factor analysis of variance was used for the overall comparison. When diversity occurred, Dunnett method was adopted for pairwise comparisons among the different dosages, positive controls and negative controls. While the variance was heterogeneous, appropriate variable conversion was done for the original data to test the homogeneity of variance. If the variance was homogeneous, the converted data was used in statistics. Otherwise, rank-sum test was used. If there were diversities in the overall comparison, Tamhane's *T*2 test was used for pairwise comparisons, and *x* ^2^ test was used for statistics of rate.

## 3. Results and Discussion

### 3.1. Mutagenicity Assay

#### 3.1.1. Ames Test

Ames test had been expensively used to help evaluate the mutagenic and carcinogenic risks for a many number of chemicals. McCann and Ames observed that a total of 5000 chemical compounds were revealed to present a mutagenic and carcinogenic risk, as determined by the Ames test [23]. In this study, the results of Ames test are summarized in Tables 1 and 2.

As can be seen, the numbers of colonies of each group at every dose of aqueous extract of* Cyclocarya paliurus* leaves in either presence or absence of S9 did not exceed twice of those of spontaneous reverse mutation group. Reversion mutation colonies did not grow with increasing dosages of aqueous extract compared to the solvent plates, indicating that no dose-response relationship was reflected. On the other hand, on all of positive plates, reversion mutation colonies grew greater than twice as frequently as on the negative plates. The results of the repeated test reflected the same pattern.

#### 3.1.2. Mouse Bone Marrow Polychromatic Erythrocyte Micronucleus Test

Mouse bone marrow micronucleus assay is internationally recognized as the standard method of detecting mutagenicity of chemicals, which is originated by Schmid in the early 1970's [24]. Generally micronucleus is derived from chromosome losing centromere or chromosome fragment after the chromosome was damaged by certain physical and chemical factors. Micronuclei incidence may reflect the extent of how chromosomes are damaged. The results of mouse bone marrow polychromatic erythrocyte micronucleus test of aqueous extract from* Cyclocarya paliurus* leaves are shown in Table 3.

Whether male or female, the incidence of micronucleus of tested group for each dosage level had no significant statistical difference compared to the negative control, so as the index of PCE/NCE (*P* > 0.05). There was no significant correlation between the micronucleus incidence and the dosage. Furthermore, each dose of the aqueous extract did not cause differences between sexes. However, there was significant difference between cyclophosphamide group and negative control group (*P* < 0.01). So it could be considered that the aqueous extracts of* C. paliurus* leaves had no marked effect on the incidence of mouse bone marrow micronucleus.

#### 3.1.3. Sperm Deformity Test in Mice

Sperm deformity test is one way to detect reproductive toxicity, including determination of the number of sperms, sperm motility, and sperm morphology. According to reports the results of three trials are consistent. Mammalian sperm abnormality test was to evaluate the reproductive toxicity of chemicals in a reliable and easy way. Singh et al. divided the sperm deformity into five kinds: acrosome defect, wedges, banana, bubble neck, and amorphous [25]. Xu et al. reported that sperm deformity through toxicity test could be divided into seven kinds: no hooks, bananas, fat head, double heads, twin tails, tail folded, and amorphous [26]. According to the experimental results, this paper reports the type of deformity with reference to Xu et al. [26].

As can be seen in Table 4, the kinds of abnormal sperm in any group were mainly no hooks, bananas, fat heads, and amorphous. The incidence of mouse sperm deformity of tested group at each dosage level had no significant difference from that of the negative control (*P* > 0.05). Significant differences could be observed between the positive controls and negative controls (*P* < 0.01). The summary of no hooks and amorphism in the three dosage groups and negative control group accounted for about 73% of the total, while in positive control group it decreased to about 60%.

### 3.2. Teratogenicity Assays

During the gestation, no signs of miscarriage, no deaths, and no gross anatomical abnormalities were observed to any pregnant rat in all groups. Summary of the pregnant rat weight data was presented in Table 5. As can be seen, the weight of pregnant rats in negative controls increased in a normal manner, so did the pregnant rats in the three dosage groups. On the 20th day, the weight gains of the three dosage groups had no significantly statistical diversity compared to the negative controls (*P* > 0.05). However, the weight growth of the pregnant rats in positive control group was slower, especially in the mid- and late-pregnancy (from 12th to 20th day), which resulted in that the weight gain was significantly lower than the negative control group on the 20th day (*P* < 0.05 or *P* < 0.01).

The effects of* C. paliurus* leaves aqueous extract on indexes of embryo toxicity were presented in Table 6. The numbers of live embryos, the weight of uterus linking to placenta, and the weight of placenta of positive controls were significantly lower than those of negative controls (*P* < 0.05 or *P* < 0.01). The incidences of dead and adsorptive fetus of positive controls were significantly higher than those of negative controls (*P* < 0.01). There were no distinguishable differences among the results of three dosages (5.0, 10.0, and 20.0 g/kg·bw) groups and negative controls (*P* > 0.05) explaining the aqueous extract of* C. paliurus* leaves had not produced obvious toxicity to the embryos of rats.

The sexuality, fetal body weight, body length, and tail length of the live fetuses were presented in Table 7. There were no distinguishable diversities of sex ratio of the fetuses among all of groups, illustrating that both aspirin and aqueous extract of* C. paliurus *have no effect on sexuality of embryos. Compared with the negative controls, the fetus body weights of positive controls were obviously lower (*P* < 0.01), and both body length and tail length were obviously shorter (*P* < 0.01). The fetal body weight, body length, and tail length had no significant differences among the three dosage groups and the negative control group (*P* > 0.05).

Stereoscope examination showed that no visible external abnormality had occurred to fetal rats of three dosage groups and negative control group, while encephalocele, exomphalocele, and anophthalmos had occurred to part of fetal rats in positive control group. Viscus examination showed that no obvious developmental deformity occurred to the fetal head, thorax, and abdominal viscera in three dosage groups and negative control group, while in positive controls, there was one fetal rat with anophthalmos, one with horseshoe kidney, one with absence of testis, and one with hydronephrosis.

The information about skeletal development of fetal rats was summarized in Table 8. The incidences of spine hypoplasia, sternum hypoplasia, pelvis hypoplasia, metacarpal hypoplasia, and phalanx hypoplasia of fetal rats in positive control group were significantly higher than those in negative control group (*P* < 0.05 or *P* < 0.01). Each kind of listed skeletal dysplasia proportion was from 18% to 40%, meaning multiple skeletal dysplasia appeared in the positive controls. The skeletal hypoplasia incidence showed no significant difference among the three dosage groups and the negative control group (*P* > 0.05), and the main kinds of skeletal hypoplasia were sternum, metacarpal, and phalanx, congruously. No obvious abnormalities of libs, carpal, long bones, and occipital bones were observed in each dosage group.

All results summarized above gave detailed information about the developments of rat embryos in each group. The data about fetal body weight, body length, tail length, and skeletal development of fetal rats provided evidences about the weight gains of mother rats shown in Table 5. Compared with the negative controls, all the data from the three dosages had no statistically significant differences. It can be considered that the aqueous extract of* C. paliurus* leaves has not any teratogenic effect on the embryos of rats, even at the highest dose of 20 g/kg·bw.

## 4. Conclusions

From genotoxicity assay (including Ames test, micronucleus test, and sperm abnormality test), it was demonstrated that aqueous extract of* C. paliurus* leaves had no genotoxicity effects in the Kunming mice at the highest dosage of 5000 *μ*g/plate, 10 g/kg·bw, and 10 g/kg·bw, respectively. While from the teratogenicity assay including the weights of pregnant rats, the indexes of embryo toxicity, the sexuality, fetal body weight, body length, and tail length of the live fetuses, the external deformities and deformity in viscera, and skeletal of the live fetuses, it was revealed that aqueous extract of* C. paliurus* leaves showed negative reproductive toxicity on rats at the highest dosage of 20.0 g/kg·bw, equivalent to 150-fold of recommended human dosage. Therefore, it can be considered that* C. paliurus* leaves are safe functional additive ingredient in food industries.

## Figures and Tables

**Table 1:** The results of Ames test on* C. paliurus* leaf aqueous extract (for the first time). Data are expressed as mean ± SD.

Dose (*μ*g/plate)	TA97		TA98		TA100		TA102	
	+S9	−S9	+S9	−S9	+S9	−S9	+S9	−S9
5000	150.0 ± 13.5	147.0 ± 7.5	42.7 ± 7.5	35.3 ± 4.0	179.3 ± 17.1	162.3 ± 18.8	285.0 ± 15.1	261.3 ± 9.9
1000	149.7 ± 26.1	151.0 ± 15.1	34.3 ± 4.6	39.3 ± 7.5	169.3 ± 15.6	174.7 ± 17.5	271.3 ± 10.7	275.3 ± 15.5
200	142.3 ± 9.5	147.3 ± 25.1	32.5 ± 5.5	43.0 ± 6.9	171.7 ± 11.8	175.7 ± 7.0	268.3 ± 18.2	278.3 ± 12.7
40	141.0 ± 15.5	142.7 ± 16.0	37.3 ± 8.0	39.0 ± 4.6	162.7 ± 10.5	169.0 ± 17.7	277.3 ± 14.2	265.7 ± 13.3
8	156.3 ± 12.7	145.3 ± 18.6	40.7 ± 4.2	37.0 ± 4.4	172.3 ± 14.3	164.3 ± 12.3	279.0 ± 13.5	267.0 ± 12.3
Spontaneous reverse	145.7 ± 16.6	148.7 ± 14.4	41.7 ± 7.6	40.0 ± 5.6	173.7 ± 13.7	170.7 ± 12.2	263.7 ± 11.0	269.3 ± 23.2
Solvent controls	154.3 ± 14.8	145.7 ± 12.9	34.7 ± 5.0	37.0 ± 8.5	172.3 ± 15.0	179.7 ± 10.8	273.7 ± 22.5	267.3 ± 11.2
Positive controls	1316.3 ± 130.6	1232.0 ± 134.2	2664.0 ± 168.7	2133.3 ± 107.7	2396.7 ± 137.6	2426.3 ± 123.6	1023.3 ± 127.4	2235.3 ± 162.0

All the above data were average value of three plates.

Positive controls: TA97 + S9, TA98 + S9, and TA100 + S9 adopted 2-AF (dose: 10.0 *μ*g/plate); TA97 − S9 and TA98 − S9 adopted 9-fluorenone (dose: 0.2 *μ*g/plate); TA100 − S9 adopted NaN_3_ (dose: 1.5 *μ*g/plate); TA102 + S9 adopted 1,8-mitoxanone (dose: 50.0 *μ*g/plate); TA102 − S9 adopted MMC (dose: 0.5 *μ*g/plate).

**Table 2:** The results of Ames test on *C. paliurus* leaf aqueous extract (for the second time). Data are expressed as mean ± SD.

Dose (*μ*g/plate)	TA97		TA98		TA100		TA102	
	+S9	−S9	+S9	−S9	+S9	−S9	+S9	− S9
5000	139.3 ± 7.8	144.7 ± 10.1	39.7 ± 7.5	36.7 ± 8.1	164.7 ± 15.3	175.7 ± 18.5	266.3 ± 26.0	268.0 ± 22.6
1000	154.0 ± 14.7	149.0 ± 23.6	36.0 ± 4.0	34.0 ± 4.6	174.0 ± 19.1	168.7 ± 19.6	272.7 ± 21.1	276.0 ± 11.3
200	142.0 ± 13.9	151.0 ± 18.0	38.3 ± 4.9	41.7 ± 4.0	167.7 ± 12.5	164.3 ± 21.4	276.7 ± 14.2	266.7 ± 23.2
40	153.3 ± 14.2	148.3 ± 18.7	42.0 ± 7.8	38.7 ± 8.6	177.3 ± 23.7	167.0 ± 14.0	273.0 ± 15.1	270.0 ± 20.8
8	141.7 ± 19.2	143.7 ± 9.3	35.0 ± 5.6	42.3 ± 4.2	166.3 ± 18.9	171.0 ± 14.7	266.3 ± 14.2	270.7 ± 8.1
Spontaneous reverse	138.0 ± 15.5	148.0 ± 9.6	39.3 ± 9.3	38.0 ± 7.2	165.3 ± 19.5	178.3 ± 12.5	265.0 ± 12.5	267.0 ± 23.0
Solvent controls	152.0 ± 26.0	141.7 ± 12.5	43.7 ± 5.9	40.7 ± 4.5	173.0 ± 12.1	166.0 ± 26.1	268.3 ± 8.0	277.7 ± 18.1
Positive controls	1206.7 ± 130.5	1186.3 ± 162.2	2548.0 ± 216.7	2283.0 ± 157.4	2452.3 ± 171.5	2522.7 ± 160.1	984.0 ± 73.4	2122.7 ± 122.9

All the above data were average value of three plates ± standard deviation.

Positive controls: TA97 + S9, TA98 + S9, and TA100 + S9 adopted 2-AF (dose: 10.0 *μ*g/plate); TA97 − S9 and TA98 − S9 adopted 9-fluorenone (dose: 0.2 *μ*g/plate); TA100 − S9 adopted NaN_3_ (dose: 1.5 *μ*g/plate); TA102 + S9 adopted 1,8-mitoxanone (dose: 50.0 *μ*g/plate); TA102 − S9 adopted MMC (dose: 0.5 *μ*g/plate).

**Table 3:** Effect of* C. paliurus* leaf aqueous extract on the rate of mouse bone marrow micronucleus. Data are expressed as mean ± SD.

Sex	Dose (g/kg·bw)	Number of animals	MNPCEs/PCEs (‰)	NCEs	PCEs/NCEs
Male	10.0	5	1.0 ± 0.7	850	1.181 ± 0.079
	5.0	5	1.6 ± 0.9	871	1.152 ± 0.077
	2.5	5	0.6 ± 0.5	883	1.137 ± 0.083
	Negative	5	0.8 ± 0.8	848	1.185 ± 0.089
	Positive	5	25.0 ± 3.7	1004	0.999 ± 0.055

Female	10.0	5	1.0 ± 1.2	836	1.201 ± 0.086
	5.0	5	1.0 ± 1.0	842	1.193 ± 0.085
	2.5	5	1.4 ± 0.5	874	1.152 ± 0.108
	Negative	5	1.2 ± 0.8	850	1.180 ± 0.074
	Positive	5	26.2 ± 2.6	1010	0.992 ± 0.046

PCE: polychromatic erythrocyte; NCE: normochromatic erythrocyte; MNPCE: micronucleated polychromatic erythrocyte.

Compared to negative controls; *P* > 0.05. Compared to positive controls; *P* < 0.01.

**Table 4:** Effect of *Cyclocarya paliurus* leaf aqueous extract on the rate of mouse sperm abnormality. Data are expressed as mean ± SD.

Dose/(g/kg·bw)	Mice number	Tested sperms	Abnormal sperms	Abnormality incidence	Constituent of different abnormal sperms/%						
					Lack hook	Banana-like	Fat-head	Morphism	Folding-tail	Double-headed	Twin-tailed
10.0	5	5000	114	2.28 ± 0.41	30.7	14.9	15.8	38.6	0.0	0.0	0.0
5.0	5	5000	106	2.12 ± 0.52	33.0	7.5	17.9	41.5	0.0	0.0	0.0
2.5	5	5000	111	2.22 ± 0.31	32.4	10.8	13.5	43.2	0.0	0.0	0.0
Negative controls	5	5000	109	2.18 ± 0.53	33.0	7.3	19.3	40.4	0.0	0.0	0.0
Positive controls	5	5000	462	9.24 ± 0.90	28.6	19.5	21.0	30.3	0.0	0.0	0.6

Compared to negative controls; *P* > 0.05. Compared to positive controls; *P* < 0.01.

**Table 5:** Effect of *C. paliurus* leaf aqueous extract on pregnant rat weight. Data are expressed as mean ± SD.

		Pregnant rat weight/(g)										Weight gain (g)	
Groups/(g/kg·bw)	Number of pregnant rats	0 day		7th day		12th day		16th day		20th day			
		$\bar{x}$ ± *s* (g)	*P*	$\bar{x}$ ± *s* (g)	*P*	$\bar{x}$ ± *s* (g)	*P*	$\bar{x}$ ± *s* (g)	*P*	$\bar{x}$ ± *s* (g)	*P*	$\bar{x}$ ± *s*	*P*
Negative controls	12	260.8 ± 17.1	1.000	287.9 ± 18.7	0.820	312.5 ± 21.1	0.768	355.3.5 ± 20.5	0.120	390.2 ± 20.7	/	133.4 ± 16.7	/
5.0	12	260.6 ± 17.1		283.0 ± 17.7		304.5 ± 18.7		347.7 ± 19.2		386.7 ± 18.9	0.935	126.0 ± 20.1	1.000
10.0	12	261.7 ± 17.6		286.2 ± 16.0		309.8 ± 18.6		358.1 ± 19.7		392.1 ± 20.2	0.999	130.3 ± 16.4	1.000
20.0	12	261.3 ± 17.3		288.8 ± 20.2		306.8 ± 18.3		354.2 ± 20.0		388.4 ± 24.4	0.995	127.1 ± 15.2	1.000
Positive controls	12	261.1 ± 18		280.7 ± 21.6		302.4 ± 25.8		336.5 ± 27.4		359.7 ± 27.9	0.006	98.6 ± 26.3	0.029

**Table 6:** Effect of* C. paliurus* leaf aqueous extract on the indexes of embryo toxicity in pregnant rat. Data are expressed as mean ± SD.

Groups/(g/kg·bw)	Numbers							Dead and adsorptive Incidence/(%)		Weight/(g)					
	Pregnant rat number	Corpora lutea		Nidation		Live embryo				Ovary		Uterus linking to Placenta		Placenta	
		$\bar{x}$ ± *s*	*P*	$\bar{x}$ ± *s*	*P*	$\bar{x}$ ± *s*	*P*	$\bar{x}$ ± *s*	*P*	$\bar{x}$ ± *s*	*P*	$\bar{x}$ ± *s*	*P*	$\bar{x}$ ± *s*	*P*
Negative controls	12	13.3 ± 2.3	0.905	11.8 ± 2.1	0.690	11.6 ± 2.1	/	1.42	/	0.186 ± 0.042	0.805	66.42 ± 6.86	/	6.70 ± 1.25	/
5.0	12	13.5 ± 2.6		11.3 ± 2.4		11.2 ± 2.5	0.977	0.74	1.000	0.183 ± 0.039		63.34 ± 9.73	0.789	6.95 ± 0.92	0.970
10.0	12	13.6 ± 1.8		11.7 ± 2.3		11.4 ± 2.3	0.999	2.14	0.684	0.194 ± 0.043		65.64 ± 8.32	0.998	6.78 ± 1.34	0.999
20.0	12	13.2 ± 2.2		11.3 ± 2.6		11.2 ± 2.7	0.977	1.47	1.000	0.198 ± 0.046		64.80 ± 10.25	0.973	6.56 ± 1.66	0.997
Positive controls	12	12.8 ± 2.1		10.5 ± 2.0		7.8 ± 1.9	0.001	25.4	0.000	0.179 ± 0.040		44.43 ± 7.01	0.000	5.05 ± 1.26	0.012

**Table 7:** Effect of* C. paliurus* leaf aqueous extract on sexuality, fetal body weight, body length, and tail length of fetal rat. Data are expressed as mean ± SD.

Groups/(g/kg·bw)	Live fetus	Male/female		Fetal body weight/(g)		Fetal body length/(mm)		Fetal tail length/(mm)	
		Ratio	P	$\bar{x}$ ± *s*	P	$\bar{x}$ ± *s*	P	$\bar{x}$ ± *s*	P
Negative controls	139	67/72 (0.93)	/	3.79 ± 0.40	/	37.38 ± 1.31	/	13.80 ± 0.65	/
5.0	134	64/70 (0.91)	0.942	3.74 ± 0.39	0.727	37.15 ± 1.28	0.456	13.66 ± 0.62	0.511
10.0	137	63/74 (0.85)	0.712	3.72 ± 0.45	0.473	37.14 ± 1.34	0.409	13.72 ± 0.87	0.995
20.0	134	69/65 (1.06)	0.587	3.81 ± 0.42	0.983	37.35 ± 1.37	0.999	13.63 ± 0.70	0.314
Positive controls	94	45/49 (0.92)	0.961	3.18 ± 0.46	0.000	35.32 ± 1.70	0.000	13.00 ± 0.66	0.000

**Table 8:** Effect of *C. paliurus* leaf aqueous extract on skeletal development of fetal rats.

Groups/(g/kg·bw)	Fetal rats number	Hypoplasia incidence/(%)				
		Spine/*P*	Sternum/*P*	Pelvis/*P*	Metacarpal/*P*	Phalanx/*P*
Negative controls	71	0.0/—	11.3/—	1.4/—	11.3/—	18.3/—
5.0	71	1.4/1.000	9.8/0.785	1.4/1.000	12.7/0.796	14.1/0.494
10.0	72	0.0/—	13.9/0.637	2.8/1.000	9.7/0.763	12.5/0.336
20.0	69	0.0/—	13.0/0.748	1.4/1.000	17.4/0.301	15.9/0.710
Positive controls	50	18.0/0.001	40.0/0.000	24.0/0.000	32.0/0.005	38.0/0.016

## References

[1] Xie MY, Li L (2001). Review in studies on chemical constituents and bioactivities of *Cyclocarya paliurus*. *Chinese Traditional and Herbal Drugs*.

[2] Fang S, Wang J, Wei Z, Zhu Z (2006). Methods to break seed dormancy in *Cyclocarya paliurus* (Batal.) Iljinskaja. *Scientia Horticulturae*.

[3] Xie MY, Li L, Nie SP, Wang XR, Lee FSC (2006). Determination of speciation of elements related to blood sugar in bioactive extracts from Cyclocarya pallurus leaves by FIA-ICP-MS. *European Food Research and Technology*.

[4] Xie MY, Xie JH (2008). Review about the research on *Cyclocarya paliurus* (Batal.) Iljinskaja. *Journal of Food Science and Biotechnology*.

[5] Xie JH, Xie MY, Nie SP, Shen MY, Wang YX, Li C (2010). Isolation, chemical composition and antioxidant activities of a water-soluble polysaccharide from *Cyclocarya paliurus* (Batal.) Iljinskaja. *Food Chemistry*.

[6] Kurihara H, Fukami H, Kusumoto A (2003). Hypoglycemic action of *Cyclocarya paliurus* (Batal.) iljinskaja in normal and diabetic mice. *Bioscience, Biotechnology and Biochemistry*.

[7] Tzianabos A, Wang JY, Kasper DL (2003). Biological chemistry of immunomodulation by zwitterionic polysaccharides. *Carbohydrate Research*.

[8] Jiang ZY, Zhang XM, Zhou J, Qiu SX, Chen JJ (2006). Two new triterpenoid glycosides from *Cyclocarya paliurus*. *Journal of Asian Natural Products Research*.

[9] Tsai MC, Song TY, Shih PH, Yen GC (2007). Antioxidant properties of water-soluble polysaccharides from Antrodia cinnamomea in submerged culture. *Food Chemistry*.

[10] Xie JH, Liu X, Shen MY (2013). Purification, physicochemical characterisation and anticancer activity of a polysaccharide from *Cyclocarya paliurus* leaves. *Food Chemistry*.

[11] Xie JH, Xie MY, Shen MY (2010). Optimisation of microwave-assisted extraction of polysaccharides from Cyclocarya paliurus (Batal.) Iljinskaja using response surface methodology. *Journal of the Science of Food and Agriculture*.

[12] Xie JH, Shen MY, Xie MY (2012). Ultrasonic-assisted extraction, antimicrobial and antioxidant activity of Cyclocarya paliurus (Batal.) Iljinskaja polysaccharides. *Carbohydrate Polymers*.

[13] Birari RB, Bhutani KK (2007). Pancreatic lipase inhibitors from natural sources: unexplored potential. *Drug Discovery Today*.

[14] Zhang J, Huang N, Lu JC (2010). Water-soluble phenolic compounds and their anti-HIV-1 activities from the leaves of *Cyclocarya paliurus*. *Journal of Food and Drug Analysis*.

[15] Zhang J, Shen Q, Lu JC (2010). Phenolic compounds from the leaves of *Cyclocarya paliurus* (Batal.) Ijinskaja and their inhibitory activity against PTP1B. *Food Chemistry*.

[16] Li S, Li J, Guan XL (2011). Hypoglycemic effects and constituents of the barks of *Cyclocarya paliurus* and their inhibiting activities to glucosidase and glycogen phosphorylase. *Fitoterapia*.

[17] Xu Q, Song YJ (2004). Research status on *Cyclocarya paliurus*. *Acta Medicinae Sinica*.

[18] Perumalla AVS, Hettiarachchy NS (2011). Green tea and grape seed extracts—potential applications in food safety and quality. *Food Research International*.

[19] Rietjens IM, Slob W, Galli C, Silano V (2008). Risk assessment of botanicals and botanical preparations intended for use in food and food supplements: emerging issues. *Toxicology Letters*.

[20] Abdel-rahman A, Anyangwe N, Carlacci L (2011). The safety and regulation of natural products used as foods and food ingredients. *Toxicological Sciences*.

[21] EFSA Safety assessment of botanicals and botanical preparations intended for use as food supplements. Draft guidance document of the scientific committee. Intermediate document adopted for public consultation on 19 November. http://www.efsa.europa.eu/EFSA/DocumentSet/sc_guidance_bota_public_consult-ation,0.pdf.

[22] Morita O, Knapp JF, Tamaki Y, Stump DG, Moore JS, Nemec MD (2009). Effects of green tea catechin on embryo/fetal development in rats. *Food and Chemical Toxicology*.

[23] Ames BN, McCann J, Yamasaki E (1975). Methods for detecting carcinogens and mutagens with the Salmonella/mammalian microsome mutagenicity test. *Mutation Research*.

[24] Schmid W (1975). The micronucleus test. *Mutation Research*.

[25] Singh H, Newton D (1982). Mithramycin-and triethylene melamine-induced sperm abnormalities in Lakeview hamsters. *Environmental Mutagenesis*.

[26] Xu WA, Wang M, Huang XS, Zhu HJ, He GL (1982). Toxicity test of herbicide amiprophos (IV): sperm abnormality test in mice. *Chinese Journal of Zhejiang Medical University*.

